# Medical Device Development for Children and Young People—Reviewing the Challenges and Opportunities

**DOI:** 10.3390/pharmaceutics13122178

**Published:** 2021-12-17

**Authors:** Paul Dimitri, Valeria Pignataro, Mariangela Lupo, Donato Bonifazi, Maria Henke, Umberto M. Musazzi, Floris Ernst, Paola Minghetti, Davide F. Redaelli, Sophia G. Antimisiaris, Giovanni Migliaccio, Fedele Bonifazi, Luca Marciani, Aaron J. Courtenay, Nunzio Denora, Angela Lopedota

**Affiliations:** 1 Department of Pediatric Endocrinology, Sheffield Children’s NHS Foundation Trust & Sheffield Hallam University, Shefeld S10 2TH, UK; paul.dimitri@nhs.net; 2 Consorzio per Valutazioni Biologiche e Farmacologiche, Via N. Putignani 178, 70122 Bari, Italy; vpignataro92@gmail.com (V.P.); dbonifazi@cvbf.net (D.B.); gmigliaccio@cvbf.net (G.M.); 3 TEDDY European Network of Excellence for Paediatric Research, Via Luigi Porta 14, 27100 Pavia, Italy; mlupo@teddynetwork.net; 4 Institute for Robotics and Cognitive Systems, University of Luebeck, Ratzeburger Allee 160, 23562 Luebeck, Germany; henke@rob.uni-luebeck.de (M.H.); ernst@rob.uni-luebeck.de (F.E.); 5 Department of Pharmaceutical Sciences, Università degli Studi di Milano, Via G. Colombo, 20133 Milan, Italy; umberto.musazzi@unimi.it (U.M.M.); paola.minghetti@unimi.it (P.M.); 6 Scientific Institute IRCCS E. Medea, Bosisio Parini, 23843 Lecco, Italy; davidefelice.redaelli@gmail.com; 7 Department of Pharmacy, University of Patras, Rio Campus, 26504 Rio Patras, Greece; santimis@upatras.gr; 8 Fondazione per la ricerca farmacologica Gianni Benzionlus, Via Abate Eustasio, 30, 70010 Valenzano, Italy; fb@benzifoundation.org; 9 Translational Medical Sciences, National Institute for Health Research (NIHR) Nottingham Biomedical Research Centre, Nottingham University Hospitals NHS Trust and University of Nottingham, Derby Road, Nottingham NG7 2UH, UK; luca.marciani@nottingham.ac.uk; 10 School of Pharmacy and Pharmaceutical Sciences, Coleraine Campus, Ulster University, Cromore Road, Coleraine, Co. Londonderry, Northern Ireland BT52 1SA, UK; a.courtenay@ulster.ac.uk; 11 Department of Pharmacy-Pharmaceutical Sciences, University of Bari Aldo Moro, Via E. Orabona 4, 70125 Bari, Italy

**Keywords:** medical devices, pediatrics, 3D printing, diagnostic imaging, delivery device, patient involvement

## Abstract

Development of specific medical devices (MDs) is required to meet the healthcare needs of children and young people (CYP). In this context, MD development should address changes in growth and psychosocial maturation, physiology, and pathophysiology, and avoid inappropriate repurposing of adult technologies. Underpinning the development of MD for CYP is the need to ensure MD safety and effectiveness through pediatric MD-specific regulations. Contrary to current perceptions of limited market potential, the global pediatric healthcare market is expected to generate around USD 15,984 million by 2025. There are 1.8 billion young people in the world today; 40% of the global population is under 24, creating significant future healthcare market opportunities. This review highlights a number of technology areas that have led to successful pediatric MD, including 3D printing, advanced materials, drug delivery, and diagnostic imaging. To ensure the targeted development of MD for CYP, collaboration across multiple professional disciplines is required, facilitated by a platform to foster collaboration and drive innovation. The European Pediatric Translational Research Infrastructure (EPTRI) will be established as the European platform to support collaboration, including the life sciences industrial sector, to identify unmet needs in child health and support the development, adoption, and commercialization of pediatric MDs.

## 1. Introduction

In the last decade, there has been an exponential rise in the development of medical devices with a concomitant rise in the number of companies working in the life sciences sector. The MD market is projected to grow from an estimated USD 455 billion in 2021 to USD 658 billion in 2028 [1]. The Medical Devices Regulation 2017/745 [2] has been developed to regulate and appropriately characterize medical devices as “any instrument, apparatus, appliance, software, implant, reagent, material or other article intended by the manufacturer to be used, alone or in combination, for human beings for one or more of the following specific medical purposes:

- diagnosis, prevention, monitoring, prediction, prognosis, treatment or alleviation of disease,
- diagnosis, monitoring, treatment, alleviation of, or compensation for, an injury or disability,
- investigation, replacement or modification of the anatomy or of a physiological or pathological process or state,
- providing information by means of in-vitro examination of specimens derived from the human body, including organ, blood and tissue donations,
- and which does not achieve its principal intended action by pharmacological, immunological or metabolic means, in or on the human body, but which may be assisted in its function by such means.

Despite the rapidly increasing growth predicted in the medical device market, this sector has been dominated by the development of devices for adult healthcare with paucity in the development of medical devices for pediatric healthcare [3,4]. Legislative and regulatory changes have been made to incentivize pediatric device development, yet the number of novel devices approved for use in pediatric population over the past decade has been relatively stagnant [4]. The commercial life sciences sector may simply perceive that the pediatric medical device market as complex and relatively small, with limited commercial opportunity. Thus, MD development for children gravitates towards the repurposing of adult’s applications, on the basis of the incorrect assumption that devices can simply be made smaller in line with a child’s size, with little consideration for changes in anatomy and physiology through growth and development. In the same way that the pharmaceutical industry adopted this now outdated and incorrect concept many years ago during drug development, the medical device market is in danger of repeating these mistakes by developing medical devices for children that are inappropriate or, worse still unsafe, leading to side effects and complications.

**Scheme 1:**
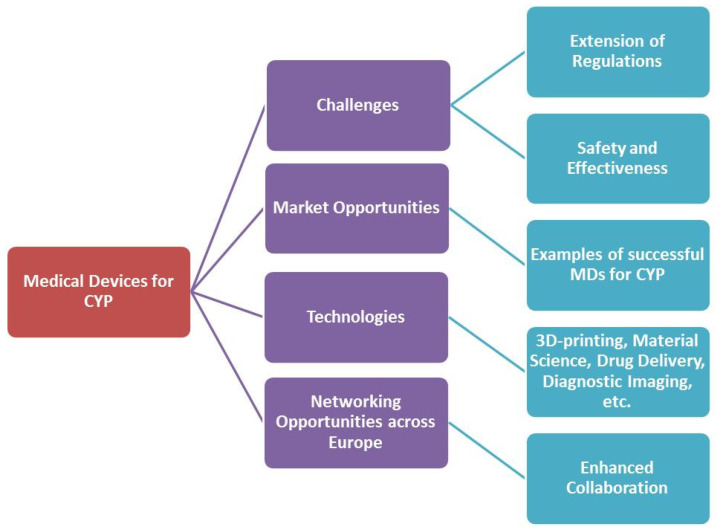
Schematic structure of this review on medical devices for children and young people.

Children represent the future, and ensuring their physical, socio-emotional, language, and cognitive development is integral to health technology development. The development of a child from term or preterm neonate to a fully mature individual relies on complex physiological, anatomical, developmental, and social changes. Understanding the inter- and intra-population differences within the pediatric subpopulations is necessary to address the existing challenges and break down some of the long-recognized barriers [5]. There is thus a clear need for research infrastructure and networks with the depth of expertise to support pediatric device development through collaboration across the life sciences sector. This review discusses the challenges and complexities in the development of MD in CYP, highlighting the need to extend current MD regulations to guarantee the safety and effectiveness of MD used in children and the value of patient and public involvement in MD device development. Potential market opportunities in this sector are discussed in this review with examples of successful MD that have been developed specifically for the pediatric population, highlighted by the diverse range of technology development in areas including 3D-printing, material science, pediatric drug delivery, and diagnostic imaging, albeit that pediatric technology development extends to a greater range of technology domains. Furthermore, the development of networking opportunities to support pediatric MD device development, particularly across Europe, has the potential to foster collaborative working and increase MD development for CYP. The outline is graphically summarized in Scheme 1.

## 2. The Complexities in the Development of Medical Devices for Children

Children undergo dynamic changes in anatomy, physiology, and development from the neonatal period through childhood into adolescence. For example, heart rate and respiratory rate reduce, and blood pressure increases as children grow (Table 1), meaning that digital platforms or wearables monitoring these parameters and others need to be capable of addressing these dynamic changes and respond appropriately to pathophysiological changes [6].

Clinical evaluation of these devices may require stratification by age, posing challenges in assessing large, stratified populations of children to appropriately power studies. Changes in anatomy will mean that versatility in device development should play an integral part in addressing the needs of the pediatric population. In this respect, technology approaches such as 3D body or facial scanning and 3D printing have led to the development of medical devices that can be adapted as anatomical changes occur with growth [8,9,10]. As children mature, there is a shift from parental dependence to independence during adolescent years, by which children move from being dependent on parental nurturing and support, to becoming young people gaining autonomy and with the ability and cognition to engage directly with medical devices without the need for parental direction. CYP with long-term conditions are surviving longer and often well into adulthood with the need to gain independent control of their condition and health, yet in Europe, less than 25% of countries allow adolescents access to health services on the basis of maturity without parental consent. Depression and anxiety disorders are among the top five causes of the disease burden, and suicide is one of the leading causes of death among adolescents, indicating that young people need access to alternative technologies to support their mental as well as physical health and wellbeing [11,12]. Thus, medical device developers need to overcome the challenge of developing devices that may initially be used by parents with their children, but in-turn meet the independent needs of adolescents with the same medical condition.

In general, there has been a significant shift in attitude to the delivery of healthcare, with a move away from a hospital-centric approach towards community or home settings with the support and integration of medical devices and a greater emphasis on self-management. This will inevitably improve the quality of life for children with long-term conditions, leaving them with more time for education and social integration, whilst reducing the number of workdays missed by parents. Improving health of CYP and the delivery of their healthcare leads to an improvement in educational attainment. In contrast, where poor school attendance and poor achievement are present, the risk of ill-health is 4.5 times higher in adulthood with 31% of school pupils aged 11–15 years reporting that their long-term condition or disability negatively impacted on their ability to participate in education [13,14]. Thus, the situational context of healthcare delivery for children, young people, and their families must be factored into the development of novel MDs to minimize disruption to their lives and limit the number of hospital attendances.

## 3. Addressing the Market Need for Pediatric Medical Devices

The need for new and innovative approaches for the development of medical devices to support CYP with acute and long-term health conditions is matched by a compelling argument to support novel technologies for prevention in childhood to ensure that our population of CYP remains healthy well into adult life. Major adult health conditions such as heart disease, stroke, hypertension, obesity, and chronic liver disease have their origins in childhood [15,16,17], yet health expenditure is typically focused on the treatment rather than prevention of these problems. This needs to be matched with funding calls focusing specifically on the development of medical devices for children to ensure targeted medical device development. To boost pediatric medical device development, pediatric child health technology networks, established to support multi-professional stakeholder collaborations involving children and their families [18,19], will accelerate the development and spread of new medical devices for pediatrics, providing a scalable offering to the commercial sector [18]. Aligned with this is the need to dispel outdated opinions that the pediatric devices market is small compared to the adult healthcare market. The global pediatric healthcare market was valued at approximately USD 11,881 million in 2018 and is expected to generate around USD 15,984 million by 2025 [20]. Europe was second to the United States in the global pediatric healthcare market in 2018, due to the increasing demand for treatments in long-term conditions and increasing healthcare infrastructure [20]. The United Kingdom is estimated to be growing rapidly over the same forecast timeframe. Germany dominated the European market with a major revenue share in 2018, due to the increasing adoption of advanced medical treatments [20]. Given the rapidly expanding pediatric healthcare market, as well as the advances in digital healthcare and data-analytics, this provides an opportunity to collect large volumes of meaningful national and international data to provide clarity about childhood growth, development, and disease in environments that will exceed traditional healthcare boundaries as the opportunities for self-management and technology-driven home-based therapies increases.

As the medical device market for pediatrics grows, industry, academics, and clinicians will be faced with the formidable challenge of how to impact upon the hard to reach and vulnerable populations. Addressing social determinants of child health and child health inequalities in large populations will be blighted by socioeconomic factors that limit technology reach. The application of novel technologies to implement change where needed most will require collaborative working between health, the life sciences industry, social care, education, and policymakers. Different processes, terminology, and cultures alongside sometimes contradictory goals and timescales can each make these collaborations a challenging venture [21,22]. Despite this, collaborative research within a triad of industrial–academic–clinical collaboration enables a fusion of diverse perspectives and expertise, often unlocking the ability to solve complex social-economic or technical problems. Driven by the emergence of combination technologies to support the development of medical devices, expertise drawn from across a range of disciplines is required [23], with universities and industry partners unlocking a range of expertise across multiple disciplines and clinicians bringing expertise relating to real-world context and integration, but importantly providing access to end-users, as either consumers or providers of healthcare.

## 4. Addressing the Regulatory Needs for Pediatric MDs

Since the early 1990s, the European Community has harmonized national regulatory frameworks to provide regulatory guidance for the classification of MD that are placed on the markets of the European Economic Area (EEA). Currently, MD designed for children must fulfil the same regulatory framework as MD for adults to enter the commercial market. Different directives and regulations have been issued to regulate MD [24], active implantable MD [25], and in vitro diagnostic MD [26]. In 2017, following the convergence of national regulatory frameworks on MD, the Regulation (EU) 2017/745 was published [2]. The regulation states specific provisions need to be in place to protect vulnerable patients, including CYP, which is fulfilled by the need to conduct clinical trials in these populations. Any clinical trial involving CYP must be able to initially demonstrate a potential benefit from their participation and must include their informed consent according to their age and maturity. However, unlike the FDA that has released guidance specifically for the development of pediatric MD assessment [27], no specific European guidance exists to manage research on MD in children or other vulnerable populations. Guidelines on clinical investigation and clinical evaluation (MEDDEV (MEDical DEVices) guideline 2.7/1 rev. 4, MDCG (Medical Device Coordination Group) guidelines from 2020-5 to 2020-13) [28,29] merely emphasize the need for establishing protocols able to assess the clinical evidence on the device efficacy and safety on the basis of the peculiarities of target population groups (e.g., pediatric populations). Similarly, few ISO standards are available for the development of MD in children [30]. In this context, it is noteworthy that article 106 of Regulation (EU) 2017/745 allows the European Commission, in consultation with the MDCG, to address these existing gaps by designating expert panels and laboratories on the basis of their up-to-date clinical, scientific, or technical expertise in the field to contribute to the development of appropriate guidance and common specifications on specific topics (e.g., clinical investigations, performance studies, biocompatibility) for specific devices in specific populations.

The following paragraphs describe some of the areas in which medical devices have been developed to meet the needs of CYP.

## 5. 3D Printing for Pediatric MD

**Figure 1:**
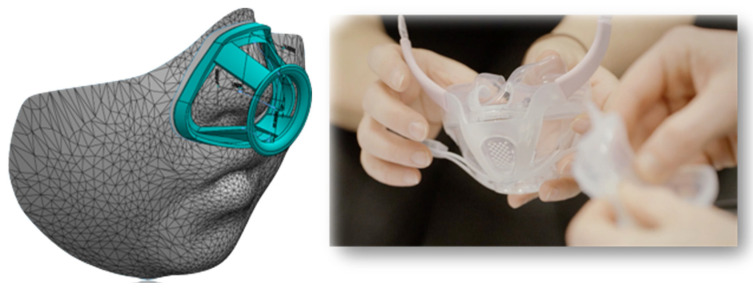
Facial mapping to develop a 3 dimensional model of a framework for a mask that will fit directly onto child’s face for non-invasive ventilation (**left**). 3D printed bespoke non-invasive ventilation mask (**right**) [8]. Pictures courtesy of Professor Heather Elphick, Sheffield Children’s NHS Foundation Trust UK.

3D printing (3DP) is a process of making three-dimensional solid objects from a digital file, by which a wide range of materials can be laid down in successive layers to form a three-dimensional object, a process referred to as additive manufacturing. Thus, 3DP provides opportunities to produce custom-made and bespoke medical products and equipment. The application of 3DP in pediatric healthcare has already been applied in specialties such as surgery, dentistry, drug delivery, orthotics and prosthetics, organs and tissues, ventilation masks, and interactive interfaces for robots and manipulators. One of the major advantages of 3DP in pediatrics is the ability to provide a bespoke product that aligns with the need for versatile manufacturing in relation to increasing body size and anatomical changes with growth. An example of this is the recent development of 3D custom-made masks for non-invasive ventilation that can accommodate the anatomical facial changes associated with growth (Figure 1) [8]. 3DP can be inexpensive, less time-consuming, and more controllable than traditional manufacturing techniques for custom-made devices—costs for molds and waste produced in machining by chip removal are reduced; milling, forging, and finishing phases are not necessary; less manual handwork is needed; and human error is reduced [31,32].

One of the earliest uses of 3DP was in the production of patient-specific anatomical models, reconstructed from the medical images derived from computerized tomography (CT scans) and magnetic resonance (MRI) for surgical planning. The same models were also be used for explaining the surgery to the patients and their families in a more effective way and for education and training [33]. Subsequent applications of 3DP in pediatric surgery include the development of customized bespoke products for implantation in growing children [34]. Orthotics and prosthetics manufacturing is one of the most active areas of 3DP technologies in CYP. Prototypes and final external devices have been developed using 3DP for interface parts (e.g., prosthetic sockets), for the whole product (e.g., ankle–foot orthoses, wrist splints, or spinal braces) [31,35] and for covering metal implantable prostheses (e.g., hip and knee prostheses or skull plates) [33]. The use of 3DP to customize prosthetic and implants provides value for both patients and healthcare professionals, as prosthetics can be produced quickly and cheaply compared to traditional manufacturing methods [36] and reduced polymer cost provides developing countries with greater access to advanced treatments [37]. Other applications of 3DP in the healthcare of CYP include dentistry, drug development, and drug delivery, creating opportunities for improving the safety, efficacy, delivery, and accessibility of medicines, as well as the creation of assistive devices for those with restricted movement [10,38,39,40].

## 6. New Materials for Pediatric Medical Devices

Novel approaches have been recently adopted to develop materials specifically for pediatric healthcare. Underpinning the development of new materials for CYP is the need to ensure that materials meet biocompatibility standards. Biocompatibility is defined as the ability of a material to perform with an appropriate host response in a specific application [41]. Biocompatibility assessment has to be done under conditions similar to the clinical setting, and usually includes in-vitro and in-vivo tests for cytotoxicity, sensitization, intracutaneous reactivity, systemic toxicity (acute), sub-chronic toxicity (sub-acute), genotoxicity, implantation (to mitigate the risk of local intolerance), and hemocompatibility [42]. For nanoparticulate components, it is critical to test interactions with biological substances at the nanoscale, where contact areas are highly expanded. Even if a particular material is known to be highly biocompatible at the macroscale, if used in a nanostructure, it should be re-tested [43]. The components of medical devices can be natural, nature-based semi-synthetic, or completely synthetic substances. After placement, MDs potentially come into contact with biological media, cells, and tissues, and may interact with them in different ways. It is of particular importance that this contact does not result in adverse reactions or toxicity. Before any MD is approved, its biological compatibility should be appropriately assessed, depending on its intended use including any potential for foreseeable misuse. The issue of “misuse” is particularly important in CYP, where age-related risks will change during growth and development. For example, a recent study reported that 33% of total pediatric MD adverse events involved ophthalmic devices, and more than 20% involved contact lenses. Greater than 40% of cases were due to non-compliant behaviors, such as wearing soft contact lenses while in shower or sleeping [44].

Although many differences apply between MDs for adult and pediatric use, no special requirements are mentioned in the European MDR (Medical Device Regulations) [2] or in relevant ISO standards, regarding safety/biocompatibility assessment of pediatric MD. Special mention of children or minors only relates to the presence of CMR (carcinogenic, mutagenic, or toxic to reproduction) and/or endocrine-disrupting substances in MD relating to specific treatments or device labelling [2]. Similarly, the FDA makes no distinction in assessing biocompatibility safety and effectiveness of MD in pediatric populations and uses the same regulatory bases and processes used to assess adult devices, but does consider MD risk assessment in relation to the age and physiological maturity of patients, the nature of the pathology, the planned duration of MD use, and exposure and the impact of the MD on growth and development [27]. In the future, regulations relating to the development of new materials in CYP need to consider their longer duration of use, the risks in the context of physiological and psychosocial maturity, and the potential differences in biocompatibility in the in-vivo environment that changes and evolves with growth and development. Many devices need to be replaced or updated as pediatric patients grow, requiring additional or different procedures to test their biocompatibility and safety, with regards to their intended duration of use, their components, and dimensions.

An example of these potential challenges is in patients with cardiac anomalies that may require use of a wide range of cardiovascular devices including synthetic heart valves, which are implanted during infancy or early childhood. As the cardiovascular anatomy is substantially modified from early infancy to adolescence, the diameters of cardiac valves increase by three times, requiring a change in valve to meet the needs of the developing patient [45]. Another application to using novel materials in CYP is microneedle (MN) technology, which provides a new opportunity for drug delivery across the skin through considerable advances in the key materials from which medical devices are manufactured, including ceramics, glasses, polymers, metals, sugars, and proteins [46]. Patch devices containing many needles less than 1 mm in length can be applied to the skin without causing bleeding or pain. Hydrogel-forming and dissolving patches can be used for drug delivery and vaccination, having potential for easy administration by children or caregivers [47].

## 7. Delivery Devices for the Administration of Pediatric Formulations

The delivery and acceptability of medicines in CYP, particularly in younger ages, is potentially challenging, with MDs now being used to improve or modify drug delivery to ensure adherence, accurate dosing, and effective delivery of pediatric formulations. Oral administration is the route preferred by adolescents and children that can ingest solid formulations such as tablets or capsules [48]. Children as young as 2 years old could swallow mini-tablets (<3 mm diameter), although swallowing tablets in children under 6 years poses challenges with a risk of non-compliance as a result [49].

**Figure 2:**
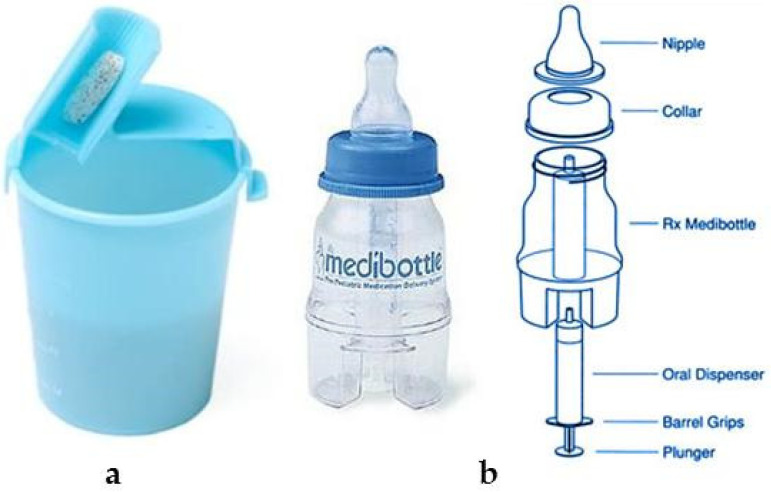
(**a**) ORALFLO™ cup; (**b**) Medibottle^®^ medication delivery devices.

For infants and babies, liquid oral formulations exist, but palatability may vary and thus they may be difficult to administer. A number of innovative solutions have been created for the administration of oral medications for CYP. For children with the problem of pill swallowing, the ORALFLO™ pill swallowing cup was developed. The patient drinks a beverage from this special cup and contextually assumes the unit dose present in a slot of the cup [50] (Figure 2a).

The X Straw^®^ device was proposed for administration of granules: the dose is in a straw that has a special control filter at the bottom. The patient dips the straw into a beverage of choice and sucks like a conventional drinking straw. During the drinking process, the filter moves up the straw and pushes the granules upwards, which are then swallowed by the patient with the beverage [51]. Modified feeding bottles such as Medibottle^®^ or pacifiers and teats have been designed for babies and infants to administer oral liquid formulations [52]. Medibottle^®^ (Figure 2b) is a traditional baby bottle with the addition of a dispenser that slides into the center sleeve of the bottle. The dose of medicine is placed in the dispenser, positioned into the bottle filled with suitable drink (milk or other), and administered to the infant by pressing the dispenser plunger while feeding [53]. Modified pacifier and teat devices position the dose of medicine in a reservoir that is attached to a hollow nipple. The baby takes the medicine either by sucking the nipple or by the caregiver compressing of the reservoir, pushing the liquid into the infant’s mouth.

**Figure 3:**
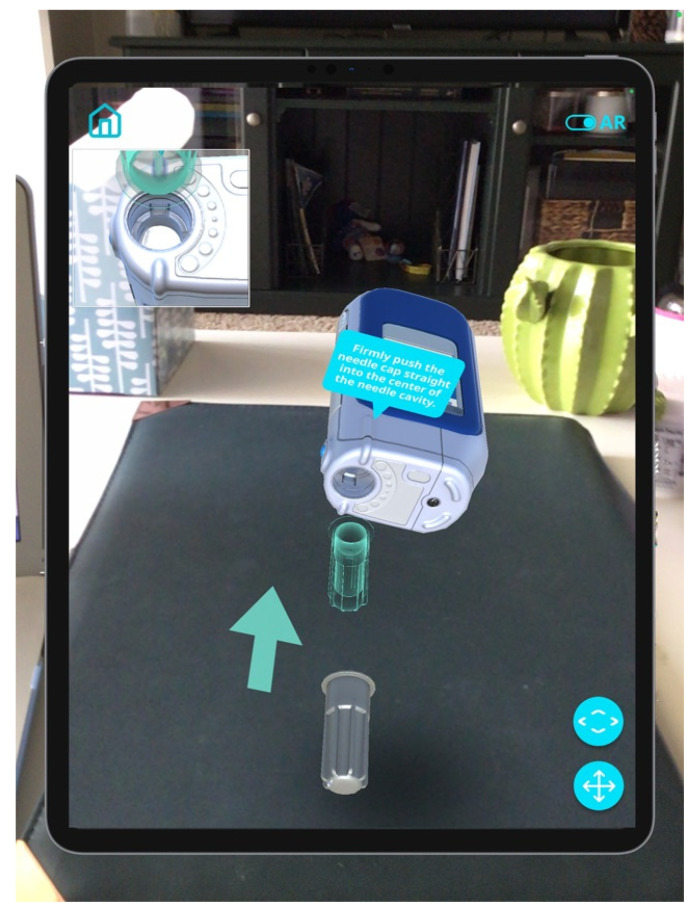
Easypod^®^ augmented reality (Merck KGaA, Darmstadt, Germany). Augmented reality is used to train caregivers and patients to load a growth hormone filled cartridge into the Easypod growth hormone injection device, with permission form [54].

Some medications cannot be given by the oral route in patients due to degradation in the digestive system, or because a more direct systemic route is required. Given the variation in body size from pre-term babies to adolescents, accurate dosing relative to weight or body surface area is required [48,52]. Medications administered by injections in children are understandably associated with anxiety and pain or discomfort, which may in turn lead to poor adherence. For medications delivered by the subcutaneous or intramuscular route, pen and auto-injector devices have been developed to support self-administration of injectable medications. These include the administration of medications such as growth hormone and insulin [52]. The Easypod^®^ device, which administers growth hormone, has extended its functionality and capabilities to incorporate functions that support training and minimize pain including augmented reality training functionality (Figure 3); electronic needle-depth adjustment; and the measurement of data relating to dose administration that can be uploaded and used as part of a digital ecosystem accessible to patients, caregivers, and healthcare professionals to improve medicine adherence [54].

Needle-free injection devices such as PharmaJet^®^, Bioject^®^, InsuJet™, Sumavel^®^, and DosePro™ are alternatives to delivering subcutaneous injections in those who are needle-phobic [55,56,57]. These devices deliver the liquid or powder formulation under high pressure through microjets that can penetrate the skin [58]. In the last decade, insulin pumps have been used in all ages as a more advanced therapy for the delivery of insulin to diabetics, providing a continuous infusion of insulin into the subcutaneous tissue, thereby eliminating the need for individual insulin injections and providing a more accurate means of modifying insulin dose with variable lifestyle demands [59]. Furthermore, attempts to link the subcutaneous continuous glucose monitoring system (CGMS) to the insulin pump could result in the development of a rudimentary external pancreas with the ability to link real-time glucose monitoring to an insulin pump to deliver the precise amount of insulin [60].

Therapy via inhalation is regarded as the best route of drug administration for the treatment of acute and chronic airway diseases in children. Pulmonary anatomy and physiology changes with age in children, with alterations in airway dimension and number [61]. Cognitive development also plays an important role in the ability to use an inhaler, and younger children are often unable to adopt an effective inhalation technique [62]. In hospital, and less frequently community/home settings, nebulizers are still widely used for inhaled therapies used, but the success of therapy in children below the age of 4 years depends on the fit and how long the facemask must be worn [63]. Younger children become upset during the long treatment times, and the treatment therefore becomes less effective. Furthermore, nebulizers, require power, need greater maintenance, and tend to be expensive and are unwieldy to be used routinely and transported. In response to the drawbacks, smaller and more portable devices (e.g., AeroNeb^®^ Go, MicroAirTM, I-Neb^®^ AAD^®^ AKITA^®^ JET) or those with shortened application time (e.g., eFlow^®^ rapid) have been developed to overcome these challenges [52,64]. Pressurized metered dose inhalers (pMDIs) and dry powder inhalers (PIs) are used as mainstay devices for the delivery of asthma drugs. To improve children’s compliance and to facilitate the use pMDIs, especially in children below the age of 4 years who have difficulties in mouth inhalation, pediatric facemask, spacers, or valve holding chambers are added on to pDMIs, examples of which include Babyhaler, Pari Vortex, Watchhaler, and Funhaler [65,66,67,68]. To measure compliance in patients, digital dose inhalers detect inhaler use and transmit data. These inhalers contain sensors that record when the medication is being administered. They are Bluetooth-enabled and can therefore be paired wirelessly with a tablet, smartphone, or computer, in order to enable automatic transfer of data from the digital dose inhaler [69].

Given the complexity of drug delivery with associated challenges in pharmacokinetics and pharmacodynamics in neonates through to adolescents, future approaches to the development of medical devices to support medicines administration in CYP need to be adaptive to age and ability, support adherence to therapy, and consider route of administration.

## 8. Diagnostic Imaging Devices for CYP

The field of diagnostic imaging in children has grown significantly in recent years [70]. Despite this, most of the imaging equipment has been traditionally developed and manufactured without specific indications for pediatric use. This is particularly important in relation to the use of ionizing radiation as younger patients are at greater risk of complications secondary to radiation exposure [71,72]. Modern dual energy techniques have reduced exposure but still have a quantifiable risk of cancer induction when use is frequent in the pediatric population [73]. In an attempt to address this issue, the FDA guidance for industry relating to premarket notifications for X-ray imaging devices encourages the inclusion of pediatric indications and provides recommendations for labeling and instruction for use [74]. The recent “Image Gently Campaign” promotes commitment safe and effective imaging in pediatrics [75], advocating the use of equipment design tailored to the unique needs of pediatric imaging. Currently, teams across Europe are working to develop MRI techniques that combine specific sequences to shorten scanning time and develop imaging techniques that use diagnostic MR and ultrasound scanning rather than CT to reduce radiation dose. Artificial intelligence has the potential to revolutionize pediatric care. Boston Children’s Hospital helped to develop and is using a decision support platform to improve the accuracy of pediatric brain scans [76]. The deep-learning tool has the ability to review thousands of images and to provide an accurate diagnosis in relation to changing brain anatomy [77]. Rapidly advancing ultrasound scan techniques are set to overcome the challenging issue of long scanning times, radiation exposure, and the need for anesthetic or sedation for younger children. Advanced ultrasound techniques constitute a suite of new technologies that employ intravascular microbubble agents to highlight perfusion abnormalities associated with various pathologies, including organ injury and residual tumors, which would otherwise be challenging to identify with conventional ultrasound. Other new ultrasound scan techniques include ultrafast Doppler to deliver high spatiotemporal resolution of flow, three- and four-dimensional technique to generate accurate spatiotemporal representation of anatomy, and high-frequency imaging to delineate anatomic structures at a resolution down to 30 μm [78]. More recently, there has been a focus on using technology to prepare children for diagnostic imaging such as MRI and CT scanning using augmented and virtual reality to familiarize children with the clinical setting and the investigative process [79,80]. Fundamental to the use of medical devices and technology to prepare children for imaging techniques is the involvement of CYP to ensure the approach is developmentally appropriate and acceptable for the intended age group.

## 9. Patient and Public Involvement in Medical Devices’ Research

Since the United Nations Convention on the Rights of the Child of 1989 [77], it is widely accepted that children have a right to express their views and to be heard in all matters that affect their lives. Article 18 of the UN Convention recognizes that children have valid insights into their well-being, valid solutions to their problems, and a valid role in implementing those solutions. Over the last decade, a Europe-wide trend toward patient-focused and patient-led research has emerged [81]. Globally, groups of healthy children and communities of pediatric patients have been established to contribute to research, on the basis of the recognition that they are “experts in their own lives” [82], as well as the fact that they provide valuable insights into service experience, research participation, and their own health and care needs [83]. Promoting the participation of young people in their healthcare produces positive outcomes, including a reduction in vulnerability and anxiety, respect, confidence, and cooperation; thus, active participation in research development and delivery should in turn engender the same results (every child has a right to be heard, UNICEF [84]). In relation to MD development for children, structured groups have emerged, including Young Persons Advisory Groups (YPAGs) [18], including the YPAG of the National Institute for Health Research (NIHR) Children and Young People Medtech Cooperative [85]. These groups have evolved on the premise that without the involvement of CYP in the development of MD in the past, products have failed to gain market traction as they are not acceptable to end-users. CYP involvement can make the design of technology better, reflect their priorities [86], and ultimately improve project success [87,88]. CYP can play an active role in MD development, from the early stage of identifying and validating unmet needs, through to proof of concept work, prototype development, formative usability evaluation, and clinical evaluation, which ultimately improves the future acceptability of medical devices. Identifying unmet needs can differ between service users and service providers: service providers will focus on the challenges faced in the delivery of healthcare, whilst service users will often focus on challenges in self-management and ways in which they can overcome personal issues relating to their health condition (working groups in the Technology Innovation Transforming Child Health (TITCH) Network, [89]). Co-creation and co-design have become established ways of integrating user-needs and views into medical device development that often include CYP. Co-creation is the joint creation of value by the organization and the end-user, allowing the end-user to co-construct the service experience to suit their context; co-design (collaborative/participative design) is the act of creating with stakeholders specifically within the design development process to ensure that the results meet their needs and are usable [90]. There are a number of frameworks developed for co-design, but one of the most well-known is the Double Diamond developed by the Design Council in 2005 [91]. The Double Diamond framework consists of two diamonds representing a process of exploring an issue or unmet need more widely or deeply (divergent thinking) and then taking focused action (convergent thinking). The diamond is split into four phases: discover—understanding the problem; define—determining the area of focus; develop—co-designing with a group of people to consider different solutions; deliver—testing solutions, rejecting those that do not work, and improving those that work (see https://www.designcouncil.org.uk/news-opinion/what-framework-innovation-design-councils-evolved-double-diamond (accessed on 30 July 2021)).

Patients of different age bands may view and adopt technology differently and not all accept technology as a potentially viable solution to unmet needs. Adolescents may reject technology that has been designed for younger children or adults due to a perceived lack of suitability, highlighting the need for evidence on “real-world” effectiveness to improve device use and adherence [92]. CYP may also express anxieties about the use of medical devices that are fundamental to the development of technology and clinical evaluation [93,94,95,96]. Recent systematic reviews highlight privacy and security issues associated with the use of mobile health applications (apps) by CYP, and CYP wanting access to safe, moderated forums to communicate with peers [91,92,93,94,95,96,97,98,99]. CYP also express concerns about new devices, limiting their ability to form a trusting therapeutic relationship with their clinical team, the permanence of information that they provide, lack of control over how their data is shared, and lack of knowledge about how healthcare professionals may respond to the information that CYP provide on digital platforms [93]. Subsequent consultation with CYP and parents, founded upon previous concerns about MD development, has led to a series of recommendations for future technology development that can be considered and integrated into the future development of pediatric MD [90]. Finding the right balance between autonomy and protection is a challenge when considering that children’s rights are situated within a larger set of parental rights and responsibilities that also focus on their best interests.

Thus, the future development of technology and the research to support evaluation of pediatric MD should always involve CYP in the identification of unmet needs, and their inclusion in developing “workable” and “real-world” products and solutions that will address their needs but that are also acceptable and usable across a broader age range.

## 10. Towards a European Infrastructure to Support Pediatric Medical Device Development

In Europe, several legislative initiatives exist for pediatric medicines [100], but none have been developed for pediatric MD. The promotion of MD development takes place at a national level but has yet to conjoin at a European scale. In 2018, the German Federal Ministry of Education and Research announced a funding program named “Small Patients, big need—medical-technical solutions for a healthcare appropriate for children” as part of the centrally recommended actions of their national strategic process of “innovations in medical technology” embedded in the Federal Government’s High-Tech Strategy. In Luebeck, the PedMedDev Hub was established to promote and improve MD for children by linking people, resources, and infrastructure, enabling strategic initiatives to accelerate safe, efficient, and cost-effective innovation. In 2014, a UK initiative was launched, called TITCH (Technology Innovation Transforming Child Health) [89], as a national collaborative network established to support the development and adoption of child health technology. This was followed by the development of the National Institute for Health Research (NIHR) Children and Young People MedTech Cooperative promoting the development of health technology for CYP across seven clinical theme areas.

The aspiration is to link up key centers and stakeholders across Europe to focus on unmet needs and harness the collaborative expertise to develop MD that will benefit European healthcare systems and that have strong commercial viability. According to this need, a new infrastructure, The European Pediatric Translational Research Infrastructure (EPTRI) [101], has been developed to support the pediatric research community, offering services, competences, and expertise in the fields of pediatric medicine discovery, pediatric biomarkers and biosamples, developmental pharmacology, pediatric medicine formulations, and MD.

By creating a network of more than 330 research units from 259 institutions distributed across 29 countries, EPTRI has identified two new and emerging areas of pediatric research that require focus from the scientific community, one of which is MD. In 2018–2019, EPTRI identified significant expertise in MD in Europe through a survey launched between April 2018 and November 2019. The survey identified a highly specialized group of 27 experts from 24 institutions of 12 different countries, mainly from the United Kingdom, Italy, and Germany. The EPTRI aim is to establish a pediatric MD network to support research and development of MD for CYP through multi-professional collaborations of European experts that benefits a large population of CYP. Areas of focus include the design and development of MD, MD validation, and end-user usability assessment, supported by tailored training for different stakeholders. The network will fundamentally be driven by a patient-centric approach to develop tailored MD for CYP.

## 11. Conclusions

There is a clear need for the life sciences industry and public sector organizations to support advances in pediatric healthcare by specifically focusing on MD developed to meet the physical, psychosocial, and functional needs of CYP. The size and diversity of the pediatric healthcare market provides the commercial life sciences sector with significant opportunities to focus development of pediatric specific MD. Whilst by no means exhaustive, this article demonstrates areas in which there has already been successful development of MD for CYP through targeted development. Regulatory and funding bodies need to keep pace with this rapidly expanding field, ensuring a patient-centric approach that safeguards younger patients. EPTRI aims to support a pan-European MD network capable of identifying unmet needs, supporting development of tailored MD and their adoption through collaboration, sharing of services, and facilitating education and training to direct the safe and effective development and commercialization of pediatric MD.

## Figures and Tables

**Table 1:** Ranges of blood pressure, pulse, and respiratory rate in relation to age of child [7].

Age	Approximate Weight (kg)	Systolic Blood Pressure (mmHg)	Heart Rate (Beats per Minute)	Respiratory Rate (Breath per Minute)
Term	3.5	60–105	110–170	25–60
3 months	6	65–115	105–165	25–55
6 months	8	65–115	105–165	25–55
1 year	10	70–120	85–150	20–40
2 years	13	70–120	85–150	20–40
4 years	15	70–120	85–150	20–40
6 years	20	80–130	70–135	16–34
8 years	25	80–130	70–135	16–34
10 years	30	80–130	70–135	16–34
12 years	40	95–140	60–120	14–26
14 years	50	95–140	60–120	14–26
17 years +	70	95–140	60–120	14–26
